# Hinokitiol reduces tumor metastasis by inhibiting heparanase via extracellular signal-regulated kinase and protein kinase B pathway

**DOI:** 10.7150/ijms.41177

**Published:** 2020-02-04

**Authors:** Yueh-Jung Wu, Wei-Jie Hsu, Li-Hsien Wu, Huei-Pu Liou, Christian Ronquillo Pangilinan, Yu-Chang Tyan, Che-Hsin Lee

**Affiliations:** 1 Department of Surgery, Kaohsiung Armed Forces General Hospital, Kaohsiung, Taiwan; 2 Department of Biological Sciences, National Sun Yat-sen University, Kaohsiung, Taiwan; 3 Department of Medical Imaging and Radiological Sciences, Kaohsiung Medical University, Kaohsiung, Taiwan; 4 Department of Medical Research, China Medical University Hospital, China Medical University, Taichung, Taiwan; 5 Department of Medical Laboratory Science and Biotechnology, Kaohsiung Medical University, Kaohsiung, Taiwan; 6 Doctoral Degree Program in Marine Biotechnology, National Sun Yat-sen University, Kaohsiung, Taiwan

**Keywords:** heparanase, tumor metastasis, hinokitiol, protein kinase B, extracellular regulated protein kinase

## Abstract

Heparanase cleaves the extracellular matrix by degrading heparan sulfate that ultimately leads to cell invasion and metastasis; a condition that causes high mortality among cancer patients. Many of the anticancer drugs available today are natural products of plant origin, such as hinokitiol. In the previous report, it was revealed that hinokitiol plays an essential role in anti-inflammatory and anti-oxidation processes and promote apoptosis or autophagy resulting to the inhibition of tumor growth and differentiation. Therefore, this study explored the effects of hinokitiol on the cancer-promoting pathway in mouse melanoma (B16F10) and breast (4T1) cancer cells, with emphasis on heparanase expression. We detected whether hinokitiol can elicit anti-metastatic effects on cancer cells via wound healing and Transwell assays. Besides, mice experiment was conducted to observe the impact of hinokitiol *in vivo*. Our results show that hinokitiol can inhibit the expression of heparanase by reducing the phosphorylation of protein kinase B (Akt) and extracellular regulated protein kinase (ERK). Furthermore, *in vitro* cell migration assay showed that heparanase downregulation by hinokitiol led to a decrease in metastatic activity which is consistent with the findings in the *in vivo* experiment.

## Introduction

Tumor cells interact with the extracellular matrix (ECM) through surface receptors or related substances or secrete proteases to degrade the matrix, which constitutes a pathway for tumor metastasis 1. Metastasis is a major problem in cancer treatment and an indicator of poor prognosis among cancer patients. Metastasis is a rather complex tumor physiological process that develops after years or even decades from diagnosis of the primary tumor 2-3. One of the enzymes responsible for ECM degradation is heparanase—an endo-β-glucuronidase that cleaves heparan sulfate (HS) side chains at a low sulfation site in the ECM. This ECM degradation process leads to some underlying biological phenomena associated with tissue remodeling and cell invasion, including inflammation, angiogenesis, and metastasis 4-5. There is an active area for tumor treatment to screen for specific heparanase inhibitors 6. Botanical preparations have been used to treat and prevent various diseases in history. Many of the anticancer drugs are plant-derived natural products and derivatives thereof, for example: the bioactive compound taxol, an alkaloid derived from Taxus brevifolia, can be used to treat malignant tumors such as breast cancer and ovarian cancer. The natural galactolipid from Crassocephalum crepidioides was shown to have anti-inflammatory activity and growth-suppressive effect against melanoma 7-8. The wood of trees in the family Cupressaceae, including the heartwood of Taiwan cypress plant or Taiwanese hinoki can purify Hinokitiol (β-thujaplicin) 9. This bioactive compound has several biological functions such as differentiation-inducing, anti-inflammatory, antibacterial, antifungal, antioxidant, and antitumor activities 10-12. Based on our previous report, hinokitiol inhibits melanogenesis in B16F10 mouse melanoma as well as inhibits vasculogenic mimicry activity of breast cancer stem/progenitor cells 13-14. However, the mechanism of hinokitiol involved in the regulation of tumor metastasis is still unclear. In this present report, we aimed to characterize the mechanism of action of hinokitiol on tumor metastasis. This study provided evidences that hinokitiol can inhibit heparanase expression via protein kinase B (Akt) and extracellular signal-regulated kinase (Erk) signaling pathways, leading to a reduction in tumor metastasis. Previous reports are consistent with the observation that treatment of hinokitiol in B16F10 cells decreases tumor cell migration 15.

## Materials and Methods

### Cell culture

Murine melanoma cells (B16F10) 16 and murine mammary carcinoma (4T1) cells 17 were maintained in 10cm culture dish with Dulbecco's Modified Eagle Medium (DMEM) containing 1% Penicillin-Streptomycin (100 units/mL penicillin and 100 μg/mL streptomycin), and 10% fetal bovine serum (FBS). Both B16F10 and 4T1 cells were incubated at 37°C, 5% CO_2_.

### Cell viability

Tumor cells were treated with varying concentrations of hinokitiol for 16 h (0, 1.25, 12.5, 125 and 1250 mM). Consequently, the cells were harvested and diluted 10-fold with trypan blue dye. Cell viability is then measured using cell counter.

### Gene transfection

B16F10 or 4T1 cells (1 × 10^6^ cells per well) were seeded in 6-well culture plates incubated for 24 h at 37°C, 5% CO2. Then, the cells were either transfected with 5 μg pCDNA 3.1 control plasmid or constitutively active AKT plasmid (Provided by Dr. Chiau-Yuang Tsai of the Department of Molecular Immunology, Osaka University, Japan) using Lipofectamine 2000 18, 19 and then incubated for another 24 h at 37°C, 5% CO2. Afterwards, the cells were washed and treated with hinokitiol (Sigma Aldrich, St. Louis, MO, USA) for 16 h and prepared for Western blotting.

### Protein extraction and quantitative analysis

B16F10 or 4T1 cells in 6-well culture plates were washed with PBS and lysed using lysis buffer (100 μl PBS containing 0.5% NP40 and 100X dilution protease inhibitor in 106 cells/well). Cells were scraped in each well and the resulting suspension was collected into Eppendorf, stand in ice for 30 min and centrifuged at 12000 rpm for 10 min, 4℃. The supernatant is then subjected to further analysis or stored at -80℃. Protein contents was determined using PierceTM BCA Protein Assay (Pierce Biotechnology, Rockford, IL, USA).

### Western blot analysis

Proteins of about 40-80 μg/mL were fractionated on SDS-PAGE, and then transferred onto nitrocellulose membranes (Pall Life Science, Glen Cove, NY, USA). Then, the membranes were probed with antibodies against heparanase (Abcam, Cambidge, UK), Akt (Santa Cruz Biotechonoly Inc., Santa Cruz, CA, USA), Erk (GeneTex International Corporation, Taiwan,), phosphorylation -Akt (Santa Cruz Biotechonoly Inc.,), phosphorylation-Erk (Merk Millipore, Germany), mTOR (Cell Signaling, Danvers, MA, USA) or β-actin (Sigma-Aldrich). The secondary antibodies was used the horseradish peroxidase-conjugated anti-rabbit IgG or anti-mouse IgG (Jackson, West Grove, PA, USA). The enhanced chemiluminescence system (Advansta, San Jose, CA, USA) was used to observe the signals. The signals was quantified by using the ImageJ software (rsbweb.nih.gov/ij/) 20, 21.

### Wound healing assay

Two-well culture-inserts was place in a 12-well plate, seeded with 2 × 10^5^ tumor cells (70 μl per well of cell solution) and incubated overnight for 37°C, 5% CO_2_. Hinokitiol (1250 nM) treatment or control (0 nM) treatments were done post-preincubation. Cell migration was assessed, and photomicrographs were taken at 0, 6, and 12 h after the addition of hinokitiol to observe the wound healing phenomenon 30.

### Transwell assay

Transwell inserts (Merk Millipore) were placed in a 24-well plate, seeded with 2× 10^5^ cells in the upper chamber (200 μl of cell solution per chamber), and added with 500 μl of FBS into each well. Traswell culture was then incubated at 37℃ with 5% CO_2_ for 24hr. Afterwards, each well were treated with hinokitiol (1250 nM) or control (0 nM) for 16 h. The cells that have penetrated the membrane were fixed with formaldehyde for 3 min, stained with DAPI (Sigma-Aldrich, St. Louis, MO, USA) for 1 min, and finally, the cells were observed and counted under a fluorescent microscope.

### Animal study

C57BL/6 mice and BALB/c mice were purchased from the National Laboratory Animal Center of Taiwan. The Laboratory Animal Care and Use Committee of the National Sun Yat-sen-University approved the animal studies ((permit number: 10624).

### Tumor treatment and survival analysis

C57BL/6J mice were inoculated with 1250 nM hinokitiol-treated or untreated B16F10 via the tail vein. Likewise, BALB/c mice were also treated with 1250 nM hinokitiol-treated or untreated 4T1. Mice mortality was recorded after tumor inoculation.

### Tumor weight and histochemical staining

C57BL/6J or BALB/c mice were inoculated with hinokitiol-treated or untreated tumor cells as already described. After 15 days, mice were sacrificed and dissected to observe lung tumor and record tumor weight. Lung tissue samples were fixed in 3.7% formaldehyde and embedded in paraffin for sectioning and histochemical staining. Tissue sections were then microscopically examined for the presence of tumor nodules.

### Statistical analysis

Image data were quantified using Image-j software. All experimental data were analyzed and presented as mean ±SD or standard deviation. The difference among the means were calculated using Student t-test of SigmaPlot statistical software. A p-value that is less than 0.05 was considered to be statistically significant.

## Results

### Cell Viability and heparanase expression in B16F10 and 4T1 following hinokitiol treatment

**Figure 1:**
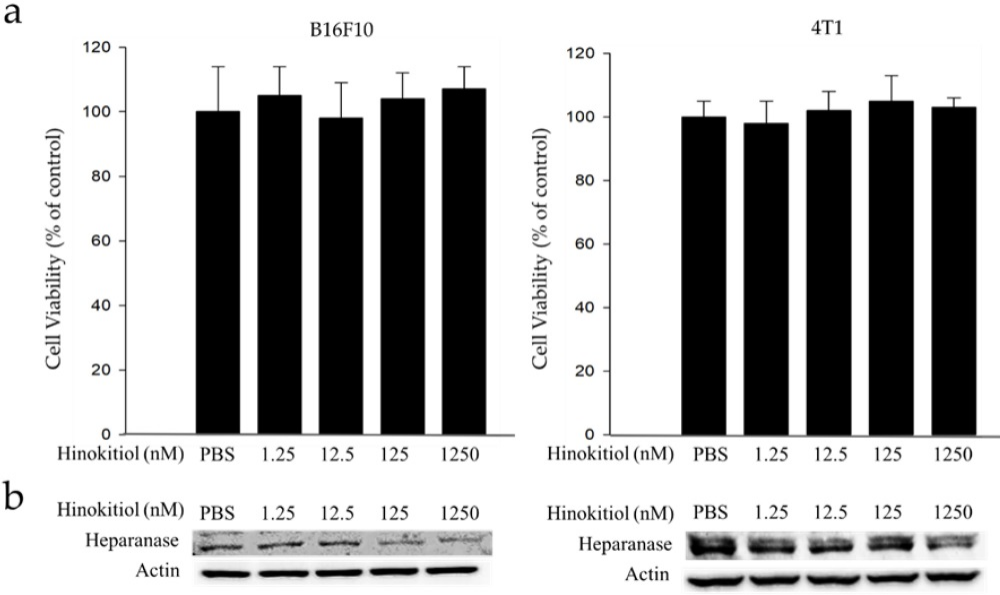
** Effects of hinokitiol on cell viability and heparanase expression in B16F10 and 4T1 cells.** Cells (5 × 10^5^ cells/well) were placed onto plates and incubated at 37 °C for 24 h and then treated with 0, 1.25, 12.5, 125 and 1250 nM hinokitiol for 16 h. (a) Cell viability was measured using cell counter after the cells were harvested and stained with trypan blue; (b) The Western blot analysis was used to detect the expression of heparanase.

Mouse B16F10 melanoma cells and 4T1 mammary carcinoma cells were used to investigate the anticancer activity of hinokitiol. This experiment determined the suitable concentration for hinokitiol that can affect tumor cell biological activities (i.e. growth, proliferation, differentiation and mobility) without harming the cells because excessive concentration may lead to direct cell death, including those with normal function. Thence, we need to find the most suitable concentration so it can achieve the benefit of anticancer effect as described in the literature 10-14. The cell survival and heparanase content of B16F10 and 4T1 cells treated with hinokitiol are shown in Figure 1. We found out that hinokitiol did not elicit substantial effects on cell viability, indicating that the dosage of hinokitiol between 0-1250 nM have no significant cytotoxicity effect against the cell lines tested (Fig. 1a). Furthermore, we evaluated the effects of hinokitiol on heparanase expression. As previously mentioned, heparanase is an important enzyme involved in the induction of tumor metastasis 4-5. The expression of heparanase in tumor cells was significantly downregulated after hinokitiol treatment as shown in Fig. 1b. The results demonstrated that hinokitiol could reduce heparanase expression in both B16F10 and 4T1 cells in a dose-dependent manner.

### Hinokitiol inhibits tumor cell migration

**Figure 2:**
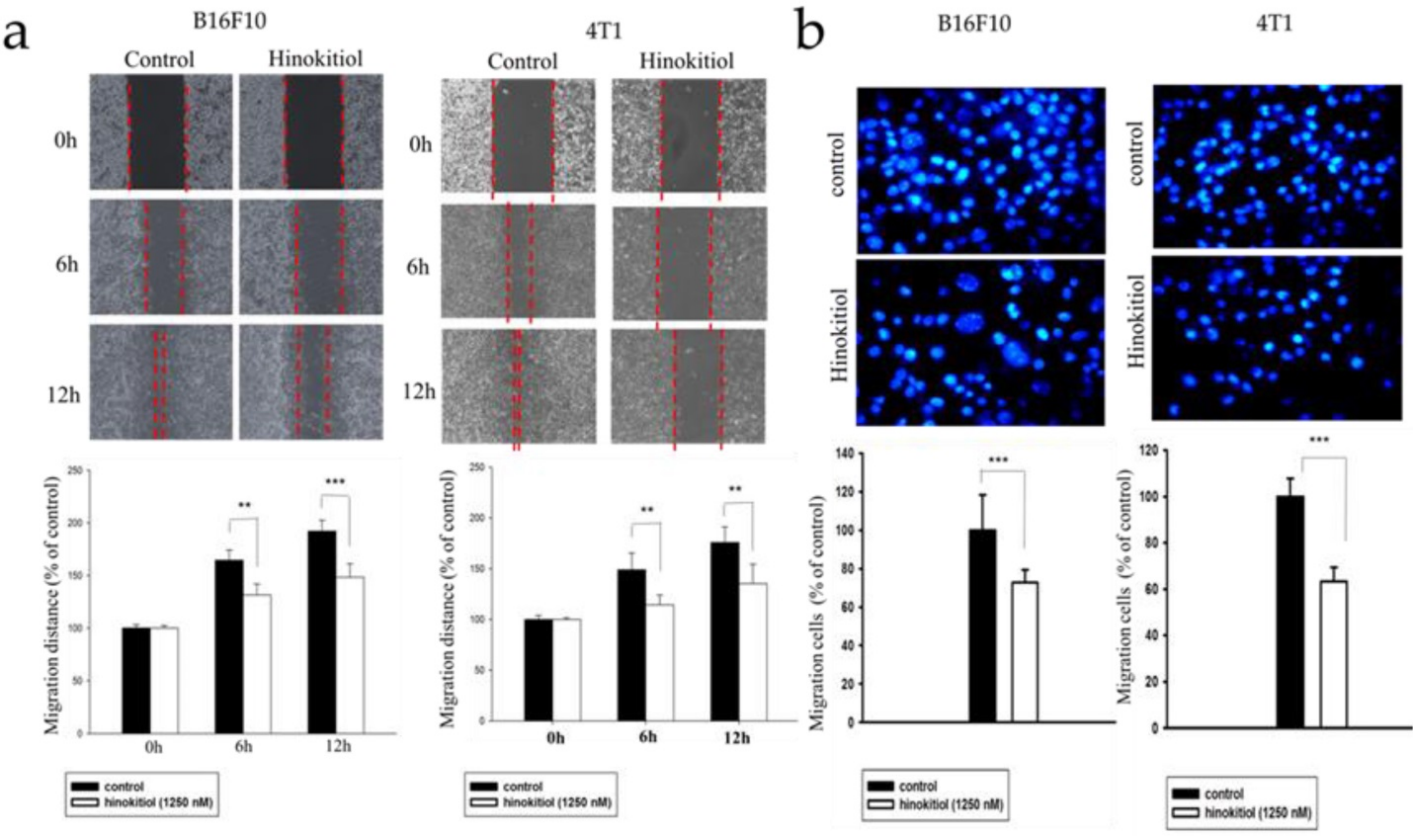
** Effects of hinokitiol on cellular migration in B16F10 and 4T1 cells.** (a) Wound healing assay. B16F10 and 4T1 cells (2×105 cells/well) were placed into 2-Well Culture-Inserts inside 12-well plate and incubated at 37℃ for 24 h. Photographs were taken at 0 h, 6 h, and 12 h after the addition of hinokitiol. Each experiment was repeated three times with similar results. In histogram, 100 present the starting point of the cell, the number above 100 is the percentage of cell migration (100%+ cell migration %) (b) Transwell assay. B16F10 and 4T1 cells (2×105 cells/well) were placed into the upper layer of a cell culture insert with a permeable membrane and incubated at 37℃ for 24 hours. Similarly, the cells were treated with hinokitiol for 16 h and then stained with 4′,6-Diamidino-2-Phenylindole (DAPI) and counted under a fluorescence microscope (×400). Each experiment was repeated three times with similar results. (n=3, mean ±SD. * p < 0.05; ** p < 0.01; *** p < 0.001)

Heparanase cleaves the heparan sulfate (HS) side chains to cause degradation of the ECM and allows cells to pass through the loosened ECM and basement membrane thereby gaining access to the lymphatic system or vascular circulation 4,5,22. Since hinokitiol inhibited heparanase expression (Fig. 1), we predicted that hinokitiol can reduce tumor cell migration. First, we utilized wound healing assay to observe migration distance of B16F10 and 4T1 tumor cells following treatment with 1250 nM hinokitiol. Cell migration distance of B16F10 and 4T1 cells was significantly decreased upon the addition of hinokitiol compared with the PBS control groups (Fig. 2a). In order to verify the phenomenon of wound healing was not caused by cell proliferation, we performed Transwell assay to examine and validate the cell migration capability of the tumor cells. As expected, the migration of B16F10 and 4T1 tumor cells were significantly reduced after a hinokitiol treatment in the Transwell assay, as seen in Fig. 2b. These results demonstrated that hinokitiol can decrease both mobility and invasion; hence, can be used to prevent the migration process.

### Hinokitiol reduced heparanase expression through protein kinase B (AKT) and extracellular signal-regulated kinase (Erk) signaling pathways

**Figure 3:**
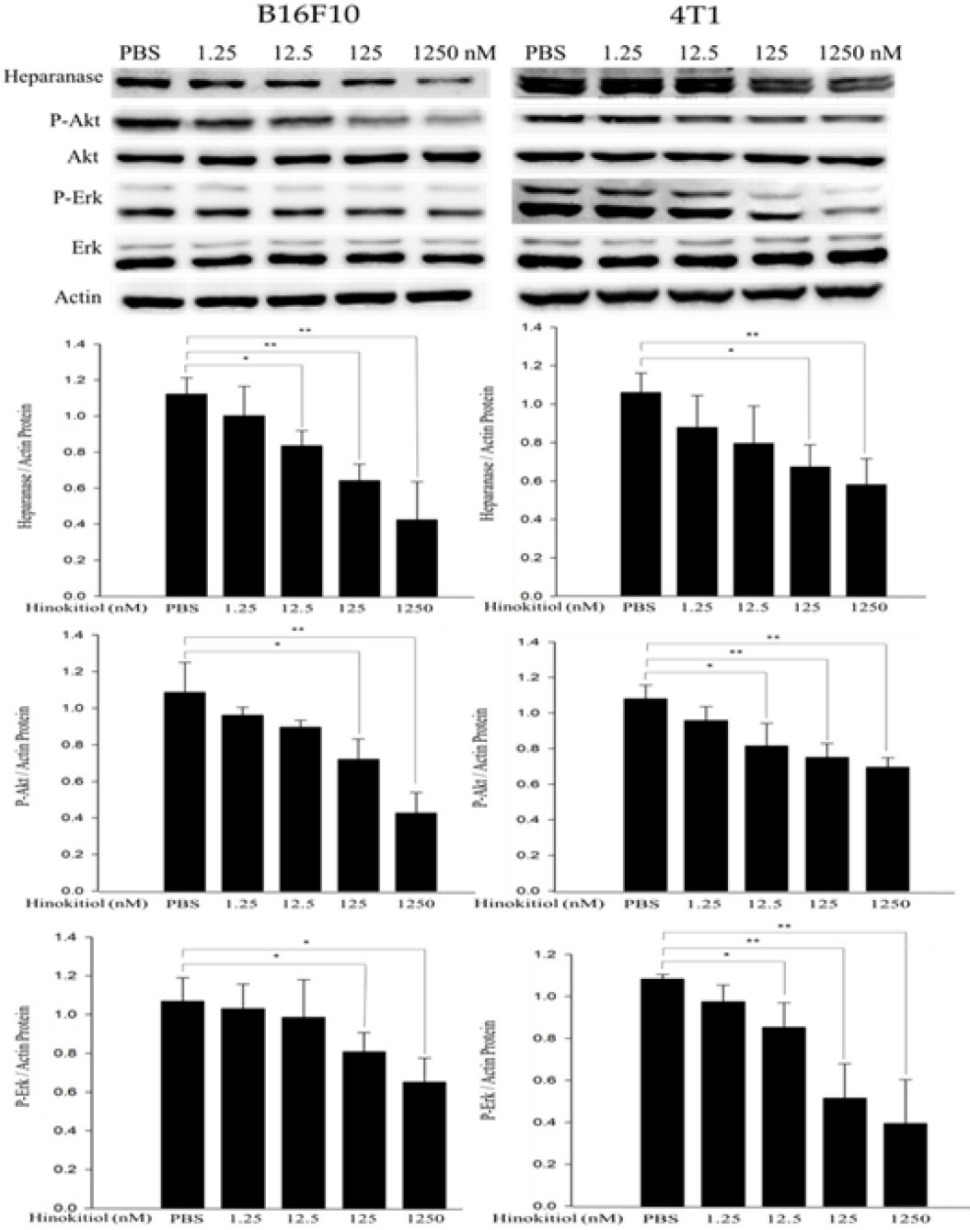
** The expression levels of heparanase and phosphorylation Akt/Erk after hinokitiol treatment.** B16F10 and 4T1 cells were treated with hinokitiol at the concentration of 0-1250 nM for 16 h. The protein expression was determined by immunoblotting. The values show the protein expression normalized to β-actin. The Western blotting assay was repeated three times with similar results. (n=3, mean ±SD. * p < 0.05; ** p < 0.01; *** p < 0.001)

The abnormal activation of cancer-promoting pathways, Akt and Erk, is associated with the process of tumor proliferation, invasion and metastasis. In addition, previous research also has mentioned that heparanase is strongly correlated with the phosphorylation of Akt and Erk pathways 23, 24. Therefore, we conduct Western blot analysis to explore whether hinokitiol can inhibit heparanase through Akt and Erk pathway. As previously mentioned, based on Fig. 1, we know that hinokitiol possessed the ability to reduce heparanase expression. In this part, B16F10 and 4T1 cells were incubated for 16 h with 0, 1.25, 12.5, 125 and 1250 nM concentrations of hinokitiol. After the treatment, cells were lysed and the phopho-Akt and phospho-Erk expression levels were measured by Western blotting (Fig. 3). The results of the Western blot analysis showed that the phosphorylation of Akt and Erk were reduced in the hinokitiol-treated group compared with PBS control group. The results suggest that hinokitiol can significantly suppress heparanase expression as well as downregulate P-Akt and P-Erk which indicate that hinokitiol may inhibit heparanase through these two pathways (Fig. 3).

### Hinokitiol elicits suppressive effect on protein expression in tumor cells bearing constitutively active-phosph-protein kinase B (P-Akt) and resveratrol-induced phosph- extracellular signal-regulated kinase (P-Erk)

Resveratrol supports the Erk signaling pathway in an activated state and can cross the blood-brain barrier 25. Therefore, in order to confirm whether hinokitiol negatively regulates heparanase through Erk pathway, we used 4 μg/mL resveratrol to induce Erk phosphorylation prior to hinokitiol treatment and then observe whether heparanase will increase Erk phosphorylation levels. Additionally, we explored Akt pathway for our experiment. We transfected tumor cells with constitutively active Akt plasmid to increase Akt transcription and phosphorylation and then observe whether heparanase will upregulate because of high phospho-Akt expression levels. Subsequently, we treated the Akt transfected-tumor cells with hinokitiol to examine the changes in heparanase expression and Akt phosphorylation.

**Figure 4:**
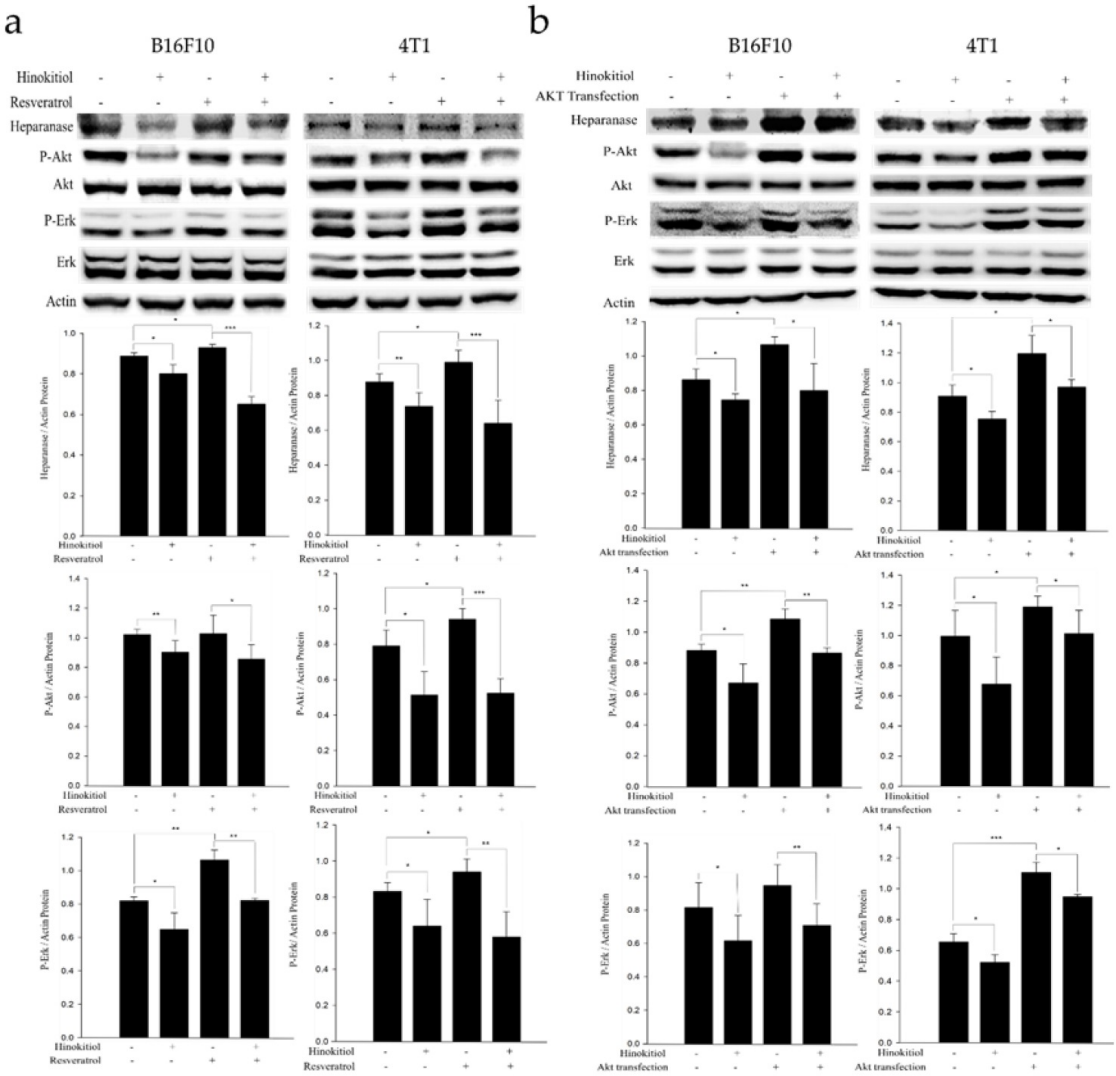
** Hinokitiol downregulates heparanase through Erk and Akt pathway.** The B16F10 and 4T1 cells (5 × 10^5^ cells/well) were placed into 6-well plates and incubated at 37℃ for 24 h. (a) Cells were treated with 4 μg/mL resveratrol to increase Erk phosphorylation for 2 hr and then treated with 0 or 1250 nM of hinokitiol for 16 h, cells were lysed and processed for western blot analysis. (b) Constitutively active-Akt transfection was performed to increase Akt expression for 24 h and then treated with 0 or 1250 nM of hinokitiol for 16 h, cells were lysed and processed for Western blot analysis. The Immunoblotting assay was repeated three times with similar results. The values show the protein expression normalized to β-actin. (n = 3, data are mean± SD).

In the resveratrol-induced Erk pathway, B16F10 and 4T1 tumor cells were treated with varying experimental conditions as follows: Both resveratrol and hinokitiol were not given (PBS group); treated only with hinokitiol (Resveratrol was not given); treated only with 4 μg/mL resveratrol (Treated resveratrol for 2 h and hinokitiol was not given), and 2 h stimulation with resveratrol prior to hinokitiol treatment for 16 hours. The results showed that resveratrol increased the phosphorylation of Erk and the expression profile of heparanase. Interestingly, hinokitiol also downregulated the phosphorylation of resveratrol-activated Erk and heparanase. These results confirm that hinokitiol inhibited the expression of heparanase indeed via Erk pathway (Fig. 4a). Moreover, in the Akt transfection experiment, B16F10 and 4T1 cells were treated with different experimental conditions as follows: Both transfected-Akt and hinokitiol were not given (PBS group); treated only with hinokitiol (No Akt transfection); transfection with Akt alone (Akt transfection for 24 h and hinokitiol was not given) and after 24 h Akt transfection, cells were treated with hinokitiol for 16 h. The results showed that Akt transfection not only increased the phosphorylation of Akt but also resulted to an elevated heparanase expression. Furthermore, hinokitiol also downregulated the Akt phosphorylation and heparanase expression of Akt-transfected tumor cells. These results confirm that hinokitiol inhibited the expression of heparanase even in the presence of a constitutively active Akt (Fig. 4b). The consistency of findings from the resveratrol and Akt transfection studies suggest that the suppression of Akt by hinokitiol will cause the reduction of Erk phosphorylation and the subsequent downregulation of heparanase.

### Hinokitiol reduces cell migration tumor cells bearing constitutively active-phosph-protein kinase B (P-Akt) and resveratrol-induced phosph- extracellular signal-regulated kinase (P-Erk)

From the results above, we now know that either the addition of resveratrol or Akt transfection in tumor cells enhanced phospho-Erk and phospho-Akt and increase heparanase expression after that. With that, we hypothesized that resveratrol treatment or Akt transfection will further induce migration ability of the cells and that after the addition of hinokitiol, migration will be inhibited.

**Figure 5:**
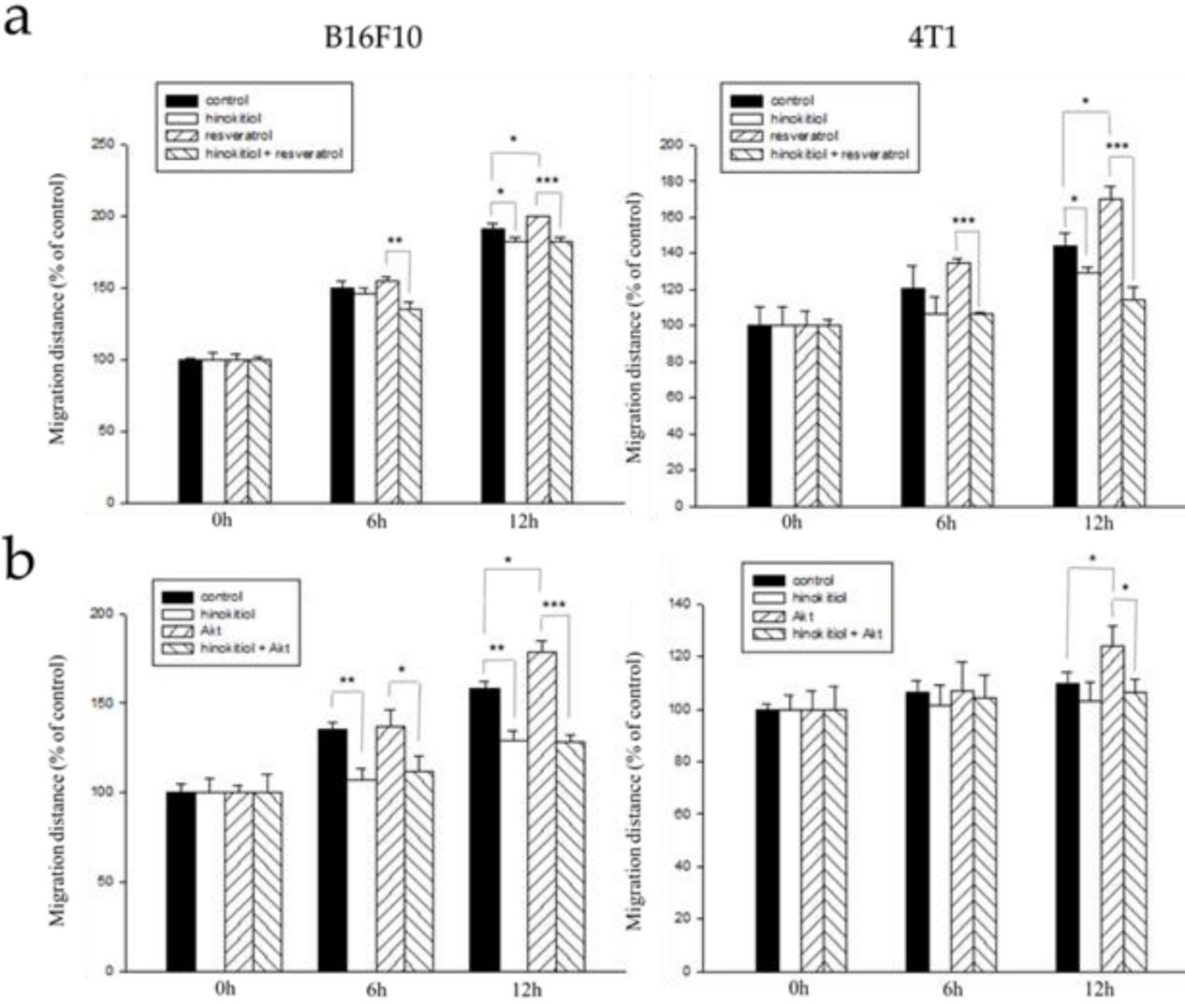
** Hinokitiol inhibits the migration through Erk and Akt pathway.** B16F10 and 4T1 cells (2 × 10^5^ cells/well) were placed into 2-Well Culture-Inserts inside a 12-well plate and incubated at 37°C for 24 h. (a) We use resveratrol to increase Erk expression for 2 h and then treat with hinokitiol. Migration distance were recorded at 0 h, 6 h, and 12 h after the addition of hinokitiol. (b) B16F10 and 4T1 cells (2 × 10^5^ cells/well) were placed into 2-Well Culture-Inserts inside a 12-well plate and incubated at 37°C for 24 h. Cells were transfected with Akt plasmid (5 μg) to increase Akt expression for 24 h and then treated with hinokitiol. Migration distance were recorded at 0 h, 6 h, and 12 h after the addition of hinokitiol. Each experiment was repeated three times with similar results. In histogram, 100 present the starting point of the cell, the number above 100 is the percentage of cell migration (100%+ cell migration %). (n=3, mean ±SD. * *p* < 0.05; ** *p* < 0.01; *** *p* < 0.001)

Here, we used the wound healing assay as described above. B16F10 and 4T1 tumor cells were treated with varying experimental conditions as follows: Group 1: Both resveratrol and hinokitiol were not given (PBS group), Group 2: Treated only with hinokitiol (Resveratrol was not given), Group 3:Treated only with 4 μg/mL resveratrol (Treated resveratrol for 2 hours and hinokitiol was not given), Group 4: 2 hour stimulation with resveratrol prior to hinokitiol treatment for 16 hours. The tumor cells were observed, and photomicrographs were taken at 0, 6, and 12 h after the addition of hinokitiol. We observed that the group 3 gained an increase in cell movement which may be accounted for by the effects of resveratrol, and after treatment with hinokitiol, migration activity was reduced. This demonstrates that hinokitiol inhibit metastasis through Erk signaling pathway (Fig. 5a). Also, we examined migration in Akt-transfected tumor cells using the same assay. B16F10 and 4T1 cells were treated with different experimental conditions as follows: Group 1: Both transfected-Akt and hinokitiol were not given (PBS group), Group 2: Treated only with hinokitiol (No Akt transfection), Group 3: Transfection with Akt alone (Akt transfection for 24 h and hinokitiol was not given), Group 4: After 24 h Akt transfection, cells were treated with hinokitiol for 16 h. Likewise, the results revealed that the group 3 gained an increase in cell movement primarily due to the effect of Akt transfection that amplified migration factors including heparanase, and after treatment with hinokitiol, migration was reduced; suggesting that hinokitiol inhibits metastasis through Akt signaling pathway (Fig 5b).

### Hinokitiol inhibits tumor metastasis and prolongs survival rate* in vivo*

**Figure 6:**
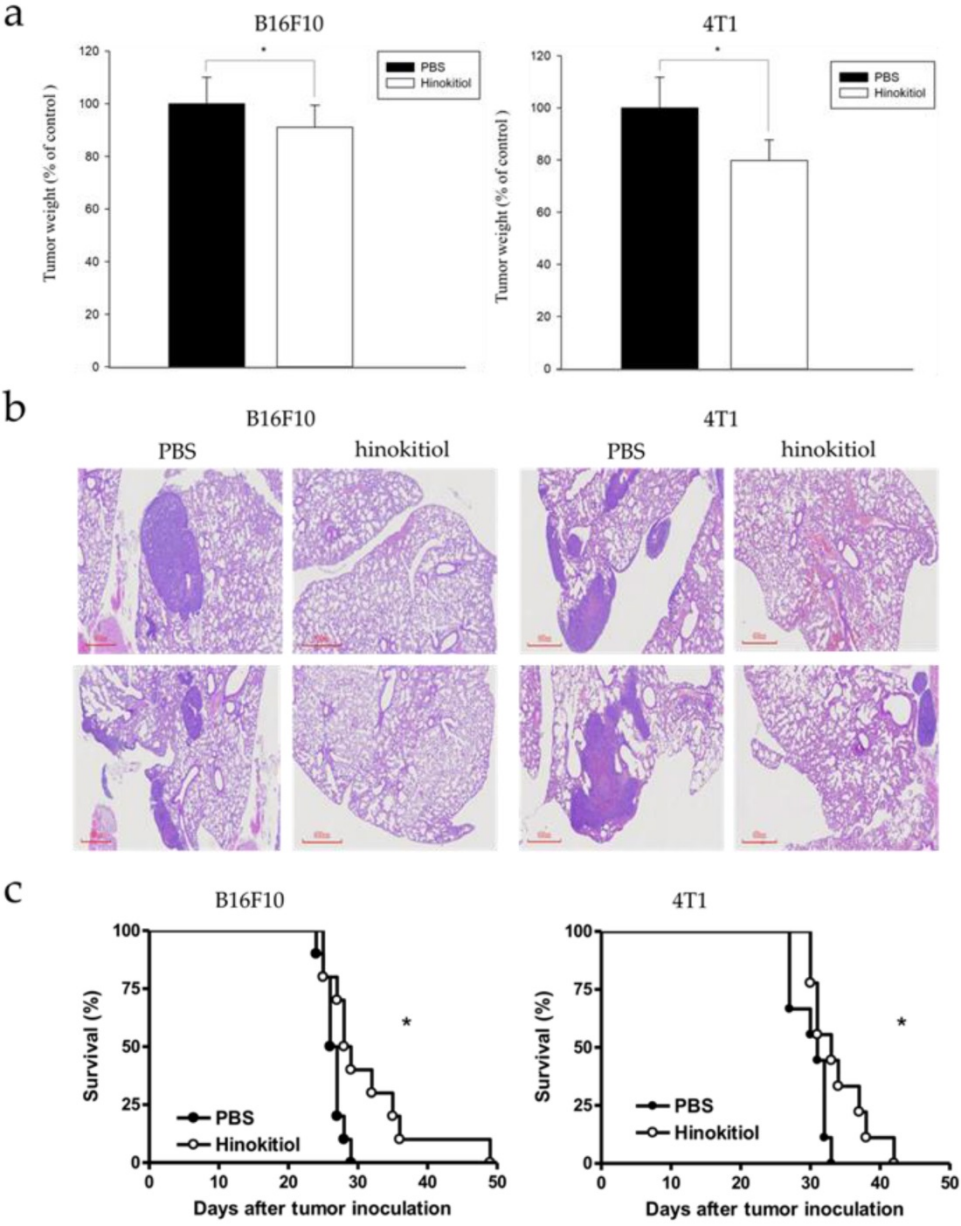
** Hinokitiol inhibited metastasis *in vivo*.** B16F10 and 4T1 cells were treated with hinokitiol or not, then cells were injected into mice via tail vein. (a) After 15 days, mice were sacrificed and the lung tumor weight were measured (n = 5-10) (b) Histological sections of lung tissue showing metastatic pulmonary tumor nodules observed at 15 days post-intravenous injection of cells (n = 5 ) (c) Recorded mice survival after tumor inoculation (n = 10-14).

Our findings above explained the physiological effects of hinokitiol *in vitro*, with emphasis on cell migration. We further confirmed the results *in vivo*. The B16F10 and 4T1 tumor cells pre-incubated with hinokitiol or not (control) were injected into mice via tail vein. After hinokitiol treatment, mice were sacrificed, and the tumor weight in the lungs was measured. The weight of lung tumors in hinokitiol-treated mice was found to be significantly lighter than that of PBS control group as shown in Fig 6a. Furthermore, histological examinations of lung tissue sections showed more tumor nodules in the mice that received B16F10 and 4T1 cells alone than those injected with cells admixed with hinokitiol (Fig. 6b). As for the mice survival rate, we also recorded the day that the animal died and found out that the lifespan was prolonged in hinokitiol-treated group (Fig. 6c). These results demonstrated that hinokitiol possessed an ability to impede tumor metastasis *in vivo* and prolonged mice survival rate.

## Discussion

Migration and invasion are vital biological behaviors of aggressive and malignant tumor cells. To date, tumor metastases, along with the development of chemoresistance and tumor relapse, remains the significant barriers to various cancer treatment modalities. To overcome this hindrance, an effective strategy to disrupt physiological activities required for tumor growth and survival is necessary. Previously, we found that hinokitiol inhibited tumor growth through the induction of autophagy 26, and apoptosis through a caspase 3-dependent pathway or cell-cycle arrest 27-28. This study used B16F10 and 4T1 cells for experiments. The B16F10, which is highly invasive, and will migrate from the primary tumor to the lungs and colonize the lungs upon intravenous (i.v.) injection. The 4T1 mammary carcinoma is highly tumorigenic and aggressive tumor cell line. It can spontaneously metastasize from a primary tumor to multiple distant locations, including lymph nodes, blood, brain, lung, liver, and bone. In this study, we identified heparanase as one of the potential targets of hinokitiol. The heparanase is involved in the hinokitiol-mediated tumor inhibition. Moreover, evidence shows that heparanase participates in tumor migration and invasion 4, 5. Hence, we hypothesized that hinokitiol drives the suppression of tumor metastasis. The expression of heparanase, wound-healing, and Transwell assay in B16F10 and 4T1 cells were significantly decreased after hinokitiol treatment. Next, we explored the signal pathway between hinokitiol and heparanase. Dr. Sheu and his colleagues, the experts in the field, found that hinokitiol inhibits tumor cell migration via blocking the phosphorylation of mitogen-activated protein kinase and p65 nuclear factor kappa B (NF-κB) 15. Our findings verified that the expression of phosphorylated Akt/Erk is decreased in hinokitiol-treated B16F10 and 4T1 tumor cells, which is consistent with the results published by other groups 15-19. We also used resveratrol and constitutively active Akt transfection to make a thorough inquiry on the association of heparanase and these two pathways. When phospho-Erk or phospho-Akt expression levels were stably activated, heparanase expression was increased considerably and after treatment with hinokitiol, not only that the Erk and Akt phosphorylation levels were decreased, but also the upregulation of heparanase protein expression was reversed. It is worth noting that we have found out that hinokitiol may behave differently in regulating protein expression in B16F10 and 4T1 cells. In 4T1 cell as shown in Figure 4b, when transfected with plasmid that constitutively expresses Akt, the phospho-Erk expression level was activated along with Akt phosphorylation. However, this knock-on effect did not appear in B16F10. Based on the findings, it can be assumed that Akt is located upstream of Erk in 4T1 tumor cell line, indicating that Akt affect Erk phosphorylation and then downregulate heparanase expression after treatment with hinokitiol. In B16F10 cell, Akt and Erk are two separate pathways regulated differently by hinokitiol. The wound healing assay also showed that cell migration was induced after P-Akt and P-Erk activation. About animal studies, less metastatic nodules and significantly lighter lung weights were recorded in hinokitiol-group, which explains the longer lifespan observed in hinokitiol-group compared with the PBS control group. Herein, we provide the similar results to demonstrate that hinokitiol reduced tumor cell migration *in vitro* and *in vivo* 15. The different target molecule (heparanase) and dose of hinokitiol were observed in this study. Hinokitiol has pleiotropic activities and appears to hold promise for the treatment of tumors.

The toxicity or carcinogenic effect of hinokitiol was not observed in animal studies. Even at a very high dose, hinokitiol was a high degree of safety for uses 28, 29. In these studies, if the highest treatment (1250 nM) uses in mice, the 22.55 μg/kg is acceptable in human. Hinokitiol exerts anti-metastasis activity through suppressing matrix metalloproteinase (MMP) 9 and 2 30. The epithelial-mesenchymal transition (EMT) transcription factor Snail expression, as known associated with metastasis, was decreased after hinokitiol treatment 31.

To sum up, this study has illustrated heparanase as a target of hinokitiol, and we believe that hinokitiol may be a potent anticancer candidate as it acts against cancer-promoting pathways namely, P-Akt and P-Erk, that drives the expression of heparanase. The suppression of P-Akt and P-Erk by hinokitiol caused a significant downregulation of heparanase in the two tumor cell lines tested which resulted to a reduced tumor metastasis both *in vitro* and* in vivo*. It is required to explore further the mechanism by which hinokitiol acts upon the cell, other than heparanase in the future. More so, it is necessary to investigate the side effects or drug damage caused by using hinokitiol. Other prospects like this investigation offer a new direction to design a more efficient way to suppress cancer. Hinokitiol can effectively downregulate tumor-induced heparanase expression in B16F10 and 4T1 cancer cell lines through the suppression of Akt and Erk signaling pathways. The result was verified using Akt transfection and resveratrol-induction. Hinokitiol is not only regulated the AKT pathway, but also regulated mitogen-activated protein kinase (MAPK ) 32, NF-κB 33, and hypoxia inducing factor-1 (HIF-1) 34. It would be interesting to test the possibility that RAC is a common regulator of cell motility mediating increased motility and invasive characteristics of cancer cells. Because Akt is dispensable for RAC regulation of actin reorganization, it has been assumed that RAC stimulate cell motility independent of Akt in mammalian cells 35. Both RAC and Akt are necessary for cell motility, and it is possible that these molecules interact functionally. This is a topic worth exploring in the future by using hinokitiol.

Furthermore, we demonstrated that hinokitiol can inhibit metastasis as demonstrated in the *in vitro* experiments that is consistent with the animal studies. Therefore, hinokitiol-mediated tumor therapy can be considered as a promising strategy in battling cancer and it merits further investigations in order to develop and design an appropriate method to suppress tumor growth and metastasis.
